# Similar Rates of Return to Play After Operative and Nonoperative Management of Physeal Nonunions and Stress Fractures of the Proximal Ulna in Athletes: A Systematic Review of 174 Patients

**DOI:** 10.1016/j.asmr.2024.101019

**Published:** 2024-10-09

**Authors:** Jacob S. Borgida, Rik J. Molenaars, Huub H. de Klerk, Bert Berenschot, Job N. Doornberg, Denise Eygendaal, Thuan V. Ly, Michel P.J. van den Bekerom

**Affiliations:** a Harvard Orthopaedic Trauma Initiative, Massachusetts General Hospital, Boston, Massachusetts, U.S.A.; b Department of Sports Medicine, Canisius-Wilhelmina Ziekenhuis, Nijmegen, the Netherlands; c Hand and Arm Research Collaborative, Department of Orthopaedic Surgery, Massachusetts General Hospital, Boston, Massachusetts, U.S.A.; d Amsterdam Shoulder and Elbow Center of Expertise (ASECE), OLVG, Amsterdam, the Netherlands; e Department of Orthopaedic Surgery, University Medical Centre Groningen (UMCG) and Groningen University, Groningen, the Netherlands; f Medical Library, Department of Research and Epidemiology, OLVG, Amsterdam, the Netherlands; g Department of Orthopaedic and Trauma Surgery, Flinders Medical Centre and Flinders University, Adelaide, Australia; h Department of Orthopaedics and Sports Medicine, Erasmus University Medical Center Rotterdam, Rotterdam, the Netherlands; i and Faculty of Behavioural and Movement Sciences, Vrije Universiteit Amsterdam, the Netherlands

## Abstract

**Purpose:**

To analyze the clinical and demographic presentation, treatment strategies, and outcomes of athletes who sustained proximal ulna stress injuries and elucidate the available evidence on management and anatomic variation.

**Methods:**

A literature search was performed on the basis of the medical databases MEDLINE/PubMed and Embase. Articles reporting on sports-related physeal injuries and stress fractures of the proximal ulna were eligible for inclusion. Outcome variables included clinical presentation, imaging assessments, management, and complications.

**Results:**

A total of 40 studies were included with 174 athletes who sustained physeal injuries or stress fractures of the proximal ulna. The age ranges of the 74 patients with physeal injuries and 100 patients with stress fractures were 11-26 years and 14-30 years, respectively. Of the studies reporting on clinical symptoms, athletes most commonly presented with olecranon tenderness to palpation and swelling. All athletes with physeal injuries were diagnosed with plain radiographs, whereas 47 athletes who underwent radiography for olecranon stress fractures required additional advanced imaging. Studies on nonoperative management reported return to play rates from 67% to 100% (median, 100% [interquartile range, 100-100%]) and 2 complications were reported (chondromalacia of the trochlear notch and occasional symptomology). One hundred twenty-six patients were treated operatively, of which failure of conservative management was reported in 15 of 40 studies, including 61 athletes. The return to play rates ranged from 67% to 100% (median, 100% [interquartile range, 100-100%]) and 20 complications were reported, most commonly hardware irritation (n = 15).

**Conclusions:**

Proximal ulna stress injuries in athletes can be managed either nonoperatively or operatively, with both showing satisfactory rates of return to play. Although operative management was more common, almost one half of these athletes failed an initial trial of nonoperative management.

**Level of Evidence:**

Level IV, systematic review of Level III-IV studies.

The proximal ulna absorbs stress during athletic activity in overhead and upper extremity weight-bearing athletes. The repetitive elbow loading in pediatric athletes, particularly in throwing sports such as baseball, can disturb normal bone maturation, resulting in physeal injuries such as fragmentation or delayed closure of the olecranon physis.^1^, ^2^, ^3^ In adolescent and skeletally mature athletes, repetitive loading of the elbow may cause “classic” stress fractures of the olecranon.^4^ These injuries are most often the result of recurrent trauma from repetitive use.^5^ Olecranon stress fractures are the most common upper extremity stress fracture in athletes and an important consideration in the young athlete with elbow pain.^6^, ^7^, ^8^, ^9^ The inclusion of both physeal injuries and various stress fracture morphologies represents the complete spectrum of stress fractures affecting the proximal ulna in athletes.^10^^,^^11^

Current management options range from nonoperative treatment and rehabilitation to operative fixation of the olecranon, but evidence-based guidelines for clinical decision making are lacking. Nonoperative treatment includes rest, splinting, platelet-rich plasma injections, bone stimulators, and physical therapy.^12^^,^^13^ For throwing athletes in particular, operative treatment is favored and undertaken using cannulated screws or tension-band systems.^14^ Often, these patients do not respond to initial nonoperative management.^14^ Timely return to play with minimal complications is important, and additional evidence is needed to optimally manage these injuries in athletes from a range of sports.

The purposes of this study are to analyze the clinical and demographic presentation, treatment strategies, and outcomes of athletes who sustained proximal ulna stress injuries and elucidate the available evidence on management and anatomic variation. We hypothesized that most athletes would undergo operative management and that the rate of return to play after injury would be high.

## Methods

This systematic review was done according to the Preferred Reporting Items for Systematic Reviews and Meta-Analyses guidelines.^15^ The protocol for this review was registered in the International Prospective Register of Systematic Reviews under the number CRD42023482325.

### Literature Search and Study Selection

With the help of a library information specialist (B.B.), a literature search was performed of the online medical databases MEDLINE/PubMed and Embase records from inception to September 14, 2023. Key words related to proximal ulna stress injuries in athletes were used. Three independent reviewers (J.S.B., R.J.M., H.H.d.K.) performed title, abstract, full-text screening, and final determination of eligibility. Reference lists were reviewed for the potential inclusion of studies omitted from the initial search. Any discrepancies about the inclusion of articles were resolved by a fourth rater (M.P.J.v.d.B.). Studies were not blinded for author, affiliation, or source.

### Inclusion and Exclusion Criteria

Articles presenting patients with a proximal ulna stress injury (physeal injury or stress fracture) as the result of athletic participation were eligible for inclusion. For the purpose of this review, the proximal end of the ulna included the greater sigmoid cavity (or trochlear or semilunar notch) and olecranon. Studies were included if they were written in English. Conference abstracts, prefaces, animal studies, and narrative or off-topic reviews were excluded. Studies focusing on fractures of the distal humerus or radial head, congenital joint disorders, and traumatic elbow fractures also were excluded.

### Data Extraction

The following data were recorded when available: number of patients, sex, age, sport, description of injury, concomitant pathology, duration of symptoms, radiologic assessment modalities used, and primary treatment modality. Relevant outcome parameters included the duration of follow-up, time to radiographic union, time to symptom resolution, time to return to play, complications, and revision surgery or surgery after initial nonoperative treatment. One study^16^ included athletes sustaining physeal injuries and stress fractures; the different groups were collected separately.

### Methodologic Quality Assessment

Two reviewers (J.B. and H.K.) independently used the Case Reports guidelines (CARE) for case reports and series that individually described patients, the Joanna Briggs Institute (JBI) critical appraisal tool for case series, and the Methodological Index for Non-Randomized Studies (MINORS) for nonrandomized cohort studies.^17^, ^18^, ^19^ In case of disagreement, the senior author (M.B.) made the final decision.

The CARE checklist involves 13 criteria, of which 8 applied to the included studies. Case series were assessed using 8 of 10 items from the JBI tool. Lastly, for the methodologic assessment of non-randomized cohort studies, the first 8 items from the MINORS checklist were used since all studies were noncomparative. All items from the 3 checklists were scored as: well described (2 points), reported but inadequate/unclear (1 point), or not reported (0 points).

### Statistical Analysis

Continuous variables are presented by indicating the full range, followed by the median value and interquartile range (IQR). Data from included studies were not pooled to avoid incorrect reporting of results because of the lack of high-level evidence.^20^ Instead, forest plots were created to visually display the data, and the I^2^ statistic was reported to explore heterogeneity among the studies.^21^ The *I*^2^ value can range between 0 and 100%, with greater values indicative of more variation between studies. All statistical analyses were conducted using Stata SE 17.0 (StataCorp LLC).

## Results

### The Search of the Literature

**Fig 1:**
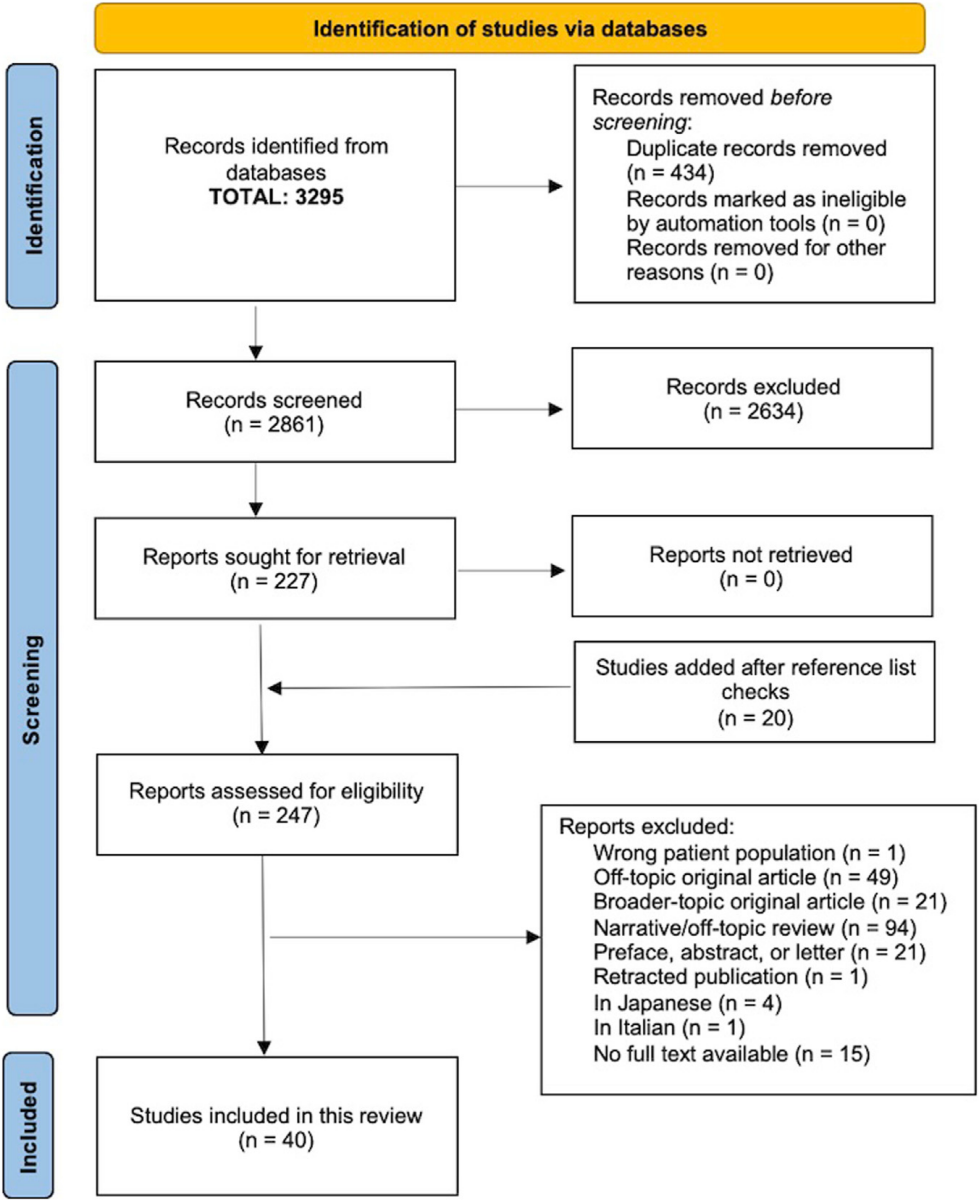
Preferred Reporting Items for Systematic Reviews and Meta-Analyses flow chart of the literature search.

The search resulted in 40 articles for synthesis (Fig 1). A library information specialist (B.B.) performed a search of MEDLINE/PubMed and Embase on September 14, 2023. In total, 3,295 articles were returned. Duplicates were identified and removed (n = 434) using the Ovid multifile search tool.^22^ The remaining 2,861 articles were screened on title and abstract using Rayyan.^23^ After review, 2,634 articles were excluded, and 227 full-text articles were screened. This resulted in 40 articles for data extraction.

Of the 40 articles included in this review, 3 articles were retrospective cohort studies (Level III evidence), 16 articles were retrospective case series (Level IV evidence), and the remaining 21 articles were case reports (Level IV evidence).

### Methodologic Quality Assessments

The mean CARE score for the 21 case reports was 13.6 ± 2.2 (range, 8-16) of a possible 16 points.^6^^,^^24^, ^25^, ^26^, ^27^, ^28^, ^29^, ^30^, ^31^, ^32^, ^33^, ^34^, ^35^, ^36^, ^37^, ^38^, ^39^, ^40^, ^41^, ^42^ The mean JBI score for the 16 case series was 13 ± 2 (range, 9-16) of a possible 16 points.^7^^,^^12^^,^^13^^,^^43^, ^44^, ^45^, ^46^, ^47^, ^48^, ^49^, ^50^, ^51^, ^52^, ^53^, ^54^ The mean MINORS score for the 3 cohort studies was 11 ± 1.7 (range, 10-13) of a possible 14 points.^55^, ^56^, ^57^ The methodologic quality assessment of the included studies, as reflected in the means, ranged from 79% to 85% of the possible points.

### Patients

Most studies were performed in the United States (27/40; 68%) or Japan (6/40; 15%). Overall, there were 174 athletes included: 162 male (93%), 5 female (3%), and 7 with an unspecified sex (4%).

Nineteen studies^7^^,^^13^^,^^16^^,^^25^^,^^27^^,^^28^^,^^35^^,^^38^, ^39^, ^40^, ^41^^,^^43^, ^44^, ^45^^,^^48^^,^^50^^,^^51^^,^^54^^,^^56^ described 74 athletes with physeal injuries (Table 1). Notably, of these studies, 12 focused on persistent physis and 1 on a process avulsion fracture. The primary sport played was baseball (44/74; 59%) followed by gymnastics (17/74; 23%). Of the studies that reported age, the range was 11 to 26 years (median, 15 years [IQR 14-17 years]). A total of 38 patients were managed both nonoperatively and operatively (51%), 31 patients were managed operatively (42%), and 5 patients were managed nonoperatively (7%). Of the studies that reported follow-up, the range was 6 weeks to 9 years (median, 1.2 years [IQR years 0.6-2.3]). Follow-up was not specified in 2 of the included studies.^45^^,^^51^

**Table 1:** Demographics and Clinical Characteristics for the Included Studies

Study	Study Design (Level of Evidence)	Country	Total Patients (Male/ Female)	Age, Mean (Range), yr	Sport	Description of Olecranon Injury	Primary Treatment	Follow-up Mean (Range), yr
Physeal injuries (n = 74)								
Yamaura et al.^54^ (2018)	CS (IV)	U.S.A.	2 (2/0) 1 patient with bilateral physeal fractures	15 (15-15)	Judo	Persistent physis	Operative	1.0
Botwin et al.^25^ (2018)	CR (IV)	U.S.A.	1 (1/0)	15	Baseball	Persistent physis	Nonoperative	0.6
Frank et al.^7^ (2017)	CS (IV)	U.S.A.	12 (12/0)	18 (14-26)	Baseball	Persistent physis	Operative	4.4 (2.6-6.1)
Clark and McKinley^27^ (2010)	CR (IV)	U.S.A.	1 (1/0)	19	Wrestling	Bilateral persistent physis	Operative	0.1
Matsuura et al.^56^ (2010)	Cohort (III)	Japan	16 (16/0)	14.3 (12-17)	Baseball	Persistent physis	Both	1.6 (1-3)
Rettig et al.^51^ (2006)	CS (IV)	U.S.A.	5 (5/0)	15 (13-17)	Baseball	Epiphyseal stress fracture nonunion	Operative	NR
Parr and Burns^16^ (2003)∗	CS (IV)	U.S.A.	2 (2/0)	14 (14-14)	Baseball; Gymnastics	Osteochondrosis	Both	0.4 (0.3-0.5)
Charlton and Chandler^44^ (2003)	CS (IV)	U.S.A.	5 (5/0)	NR (15-20)	Baseball	Persistent physis	Operative	2.7 (0.6-7.0)
Tavares^38^ (1998)	CR (IV)	U.S.A.	1 (1/0)	14.8	Baseball	Nonunion of epiphysis	Operative	1.0
Lowery et al.^48^ (1995)	CS (IV)	U.S.A.	3 (3/0)	16 (15-17)	Baseball	Persistent physis	Both	1.2 (0.5-2.0)
Walker^40^ (1995)	CR (IV)	U.S.A.	1 (1/0)	20	Weightlifter	Persistent physis	Operative	2.0
Maffulli et al.^13^ (1992)	CS (IV)	United Kingdom	10 (8/2)	14 (11-19)	Gymnastics	Physeal widening; stress fracture	Both	6.2 (1.0-9.0)
Chan et al.^43^ (1991)	CS (IV)	United Kingdom	7 (NR)†	NR	Gymnastics	Epiphysis abnormalities	Both	1.5
Weiss and Sauers^41^ (1990)	CR (IV)	U.S.A.	1 (1/0)	16	Wrestling	Process avulsion fracture	Operative	0.4
Retrum et al.^35^ (1986)	CR (IV)	U.S.A.	1 (1/0)	16	Tennis	Delayed closure of epiphysis	Nonoperative	3.0
Danielsson et al.^45^ (1983)	CS (IV)	Sweden	2 (2/0)†	13 (12-14)	Field hockey; Gymnastics	Apophysitis	Nonoperative	NR
Pavlov et al.^50^ (1981)	CS (IV)	U.S.A.	2 (2/0)	16 (16-16)	Baseball; Wrestling	Epiphysis nonunion	Operative	0.2 (0.1-0.3)
Hunter and O’Connor^28^ (1980)	CR (IV)	U.S.A.	1 (1/0)	15	Tower-diving swimmer	Traction apophysitis	Nonoperative	0.8
Torg et al.^39^ (1977)	CR (IV)	U.S.A.	1 (1/0)	16	Baseball	Nonunion of a stress fracture through the epiphyseal plate	Operative	2.3
Stress fractures (n = 100)								
Shanley et al.^57^ (2023)	Cohort (III)	U.S.A.	61 (61/0); 1 stress fracture	NR	Baseball	Stress fracture	Operative	NR
Zaremski et al.^58^ (2022)	CR (IV)	U.S.A.	1 (1/0)	15	Baseball	Stress fracture	Operative	1.0
Erickson et al.^55^ (2019)	Cohort (III)	U.S.A.	52 (52/0); 46 stress fractures	NR	Baseball	Stress fractures	Operative	>1
Brucker et al.^6^ (2015)	CR (IV)	U.S.A.	1 (1/0)	17	Baseball	Stress injury	Nonoperative	0.1
Paci et al.^49^ (2013)	CS (IV)	U.S.A.	18 (18/0)	20.4 (16.1-23.8)	Baseball	Stress fractures	Operative	6.2 (2.0-10.1)
Stephenson et al.^37^ (2012)	CR (IV)	U.S.A.	1 (1/0)	21	Baseball	Stress fracture	Operative	1.4
Fujioka et al.^46^ (2012)	CS (IV)	Japan	6 (5/1)	18 (16-21)	Baseball; Softball	Stress fractures	Operative	2.3 (2-3)
Mauro et al.^32^ (2011)	CR (IV)	U.S.A.	1 (1/0)	21	Baseball	Stress fracture	Operative	1.0
Imade et al.^29^ (2011)	CR (IV)	Japan	1 (1/0)	23	Baseball	Stress fracture	Operative	1.0
Blake et al.^24^ (2008)	CR (IV)	U.S.A.	1 (1/0)	17	Baseball	Trochlear groove stress fracture	Nonoperative	0.4
Lu et al.^30^ (2006)	CR (IV)	Taiwan	1 (1/0)	18	Baseball	Stress fracture	Nonoperative	1.0
Shinozaki et al.^36^ (2006)	CR (IV)	Japan	1 (1/0)	14	Tower-diving swimmer	Stress fracture	Nonoperative	NR
Nakaji et al.^33^ (2006)	CR (IV)	Japan	1 (1/0)	25	Baseball	Stress fracture	Operative	2.0
Burman et al.^26^ (2004)	CR (IV)	Canada	1 (1/0)	30	Baseball	Stress fracture of physis	Operative	1.5
Parr and Burns^16^ (2003)∗	CS (IV)	U.S.A.	1 (1/0)	16	Baseball	Stress fracture	Nonoperative	0.4
Schickendantz et al.^52^ (2002)	CS (IV)	U.S.A.	7 (7/0)	21.8 (18.8-29.1)	Baseball	Osseous stress injury	Nonoperative	4.0 (2.0-7.0)
Rao et al.^34^ (2001)	CR (IV)	India	1 (1/0)	20	Weightlifter	Stress fracture	Operative	0.3
Mamanee et al.^31^ (2000)	CR (IV)	U.S.A.	1 (1/0)	14	Baseball	Stress lesion	Nonoperative	1.0
Suzuki et al.^53^ (1997)	CS (IV)	Japan	2 (2/0)	19 (17-21)	Baseball	Stress fracture	Operative	0.8 (0.5-1.0)
Nuber and Diment^12^ (1992)	CS (IV)	U.S.A.	2 (2/0)	22 (21-23)	Baseball	Stress fractures	Nonoperative	0.4 (0.3-0.5)
Wilkerson and Johns^59^ (1990)	CR (IV)	U.S.A.	1 (1/0)	14	Gymnastics	Stress fracture nonunion	Operative	1.0
Hulkko et al.^47^ (1986)	CS (IV)	Finland	4 (3/1)	23 (21-28)	Javelin	Stress fractures	Both	1.5 (0.8-1.8)

CR, case report; CS, case series; NR, not reported; N/A, not applicable.

∗ Article describes patients with physeal injuries (n = 2) and osseous stress injury (n = 1).

† Patients were excluded for injuries that did not involve the olecranon or that were not sport-related (Chan et al., 1991, n = 12; Danielsson et al., 1982, n = 2).

Twenty-two studies^6^^,^^12^^,^^16^^,^^24^^,^^26^^,^^29^, ^30^, ^31^, ^32^, ^33^, ^34^^,^^36^^,^^37^^,^^46^^,^^47^^,^^49^^,^^52^^,^^53^^,^^55^^,^^57^, ^58^, ^59^ described 100 athletes with stress fractures of the olecranon (Table 1). These fractures occurred as a result of various sport participation: baseball (92/100; 92%), javelin (4/100, 4%), softball (1/100; 1%), 1/100 gymnastics (1%), 1/100 tower diving (1%), and 1/100 weightlifting (1%). Of the studies that reported age, the range was 14 to 30 years (median, 20 years [IQR 17-21 years]). A total of 81 patients (81%) with stress fractures underwent operative treatment, 15 patients (15%) were managed nonoperatively, and 4 patients (4%) were managed both nonoperatively and operatively. Of the studies that reported follow-up, the range was 6 weeks to 10 years (median 1 year [IQR, 0.7-1.5 years]). Follow-up was not specified in 2 of the included studies.^36^^,^^57^

### Clinical Presentation

Patients’ symptoms were reported in 37 studies, equating to 121 athletes. The duration of symptoms from onset to first presentation varied, with a range of 1 week to 60 months. On physical examination, reported symptoms included pain during athletic activity, olecranon tenderness to palpation, and swelling. Loss of elbow extension was reported in 9 studies^16^^,^^35^^,^^38^^,^^39^^,^^41^^,^^44^^,^^45^^,^^48^^,^^50^ on athletes with physeal injuries and in 7 studies^16^^,^^30^^,^^32^^,^^33^^,^^52^^,^^53^^,^^59^ on athletes with stress fractures.

### Imaging

**Fig 2:**
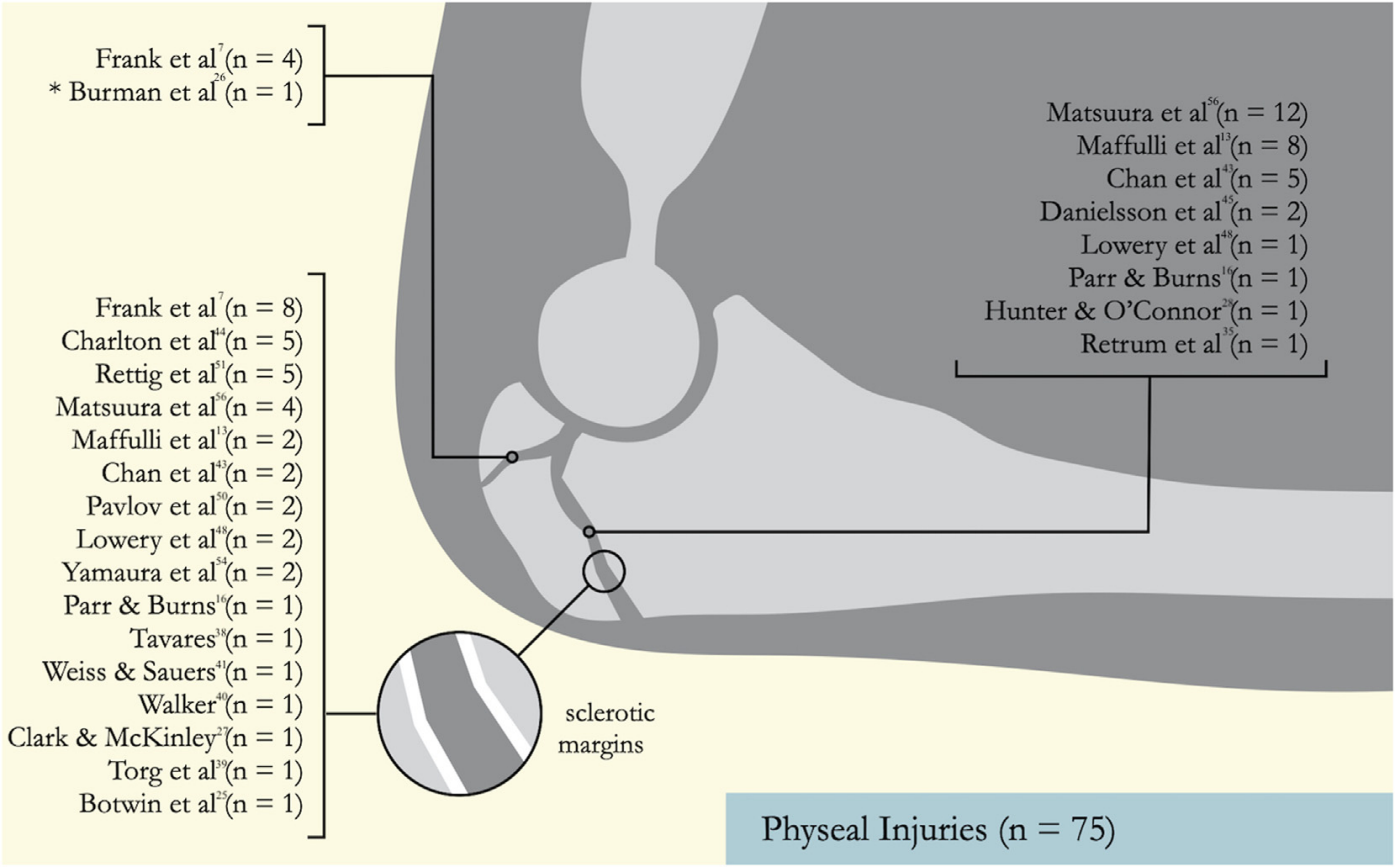
The anatomical appearance of physeal injuries (∗stress fracture that led to a physeal injury). The sclerotic margins refer to areas of increased bone density at the edges of the physis, indicating chronic stress injury. The 2 lists of papers shown in the figure refer to the anatomical locations of the injury.

**Fig 3:**
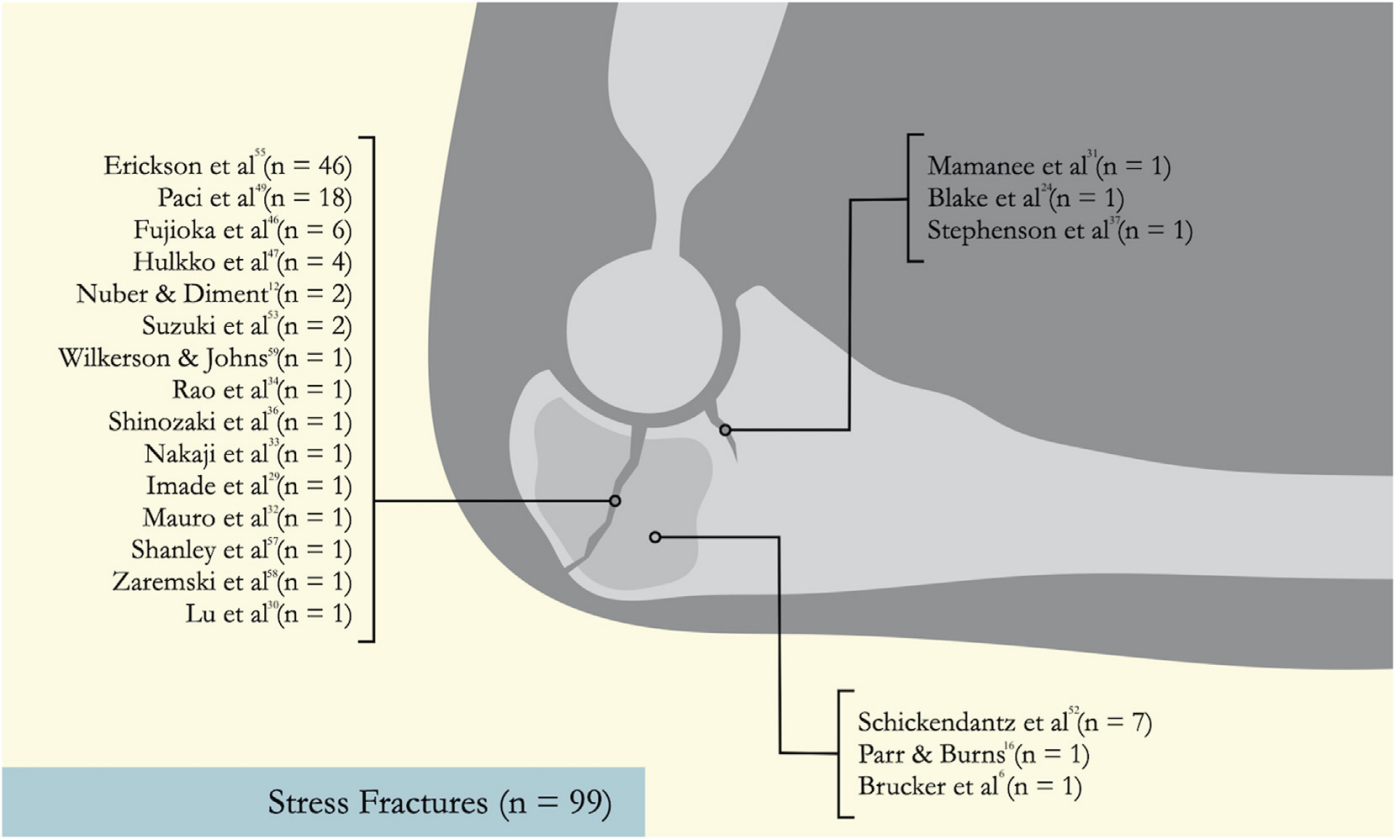
The anatomical appearance of stress fractures. The 3 lists of papers refer to the anatomical locations of the injury.

Plain radiographs were performed on all 74 athletes with physeal injuries. Three athletes underwent additional imaging with magnetic resonance imaging (MRI), and 1 athlete required a computed tomography (CT) scan.^7^^,^^25^^,^^54^ Of the studies reporting on stress fractures, 35 athletes who underwent radiography for olecranon stress fractures required additional evaluation through MRI and 2 required CT scans.^6^^,^^12^^,^^16^^,^^24^^,^^29^, ^30^, ^31^, ^32^^,^^36^^,^^37^^,^^46^^,^^49^^,^^52^^,^^58^ MRI revealed a concomitant ulnar collateral ligament (UCL) injury in 1 athlete.^58^ The variation in radiographic morphology of proximal ulna stress injuries is presented in Figures 2 and 3.

### Nonoperative Treatment

**Fig 4:**
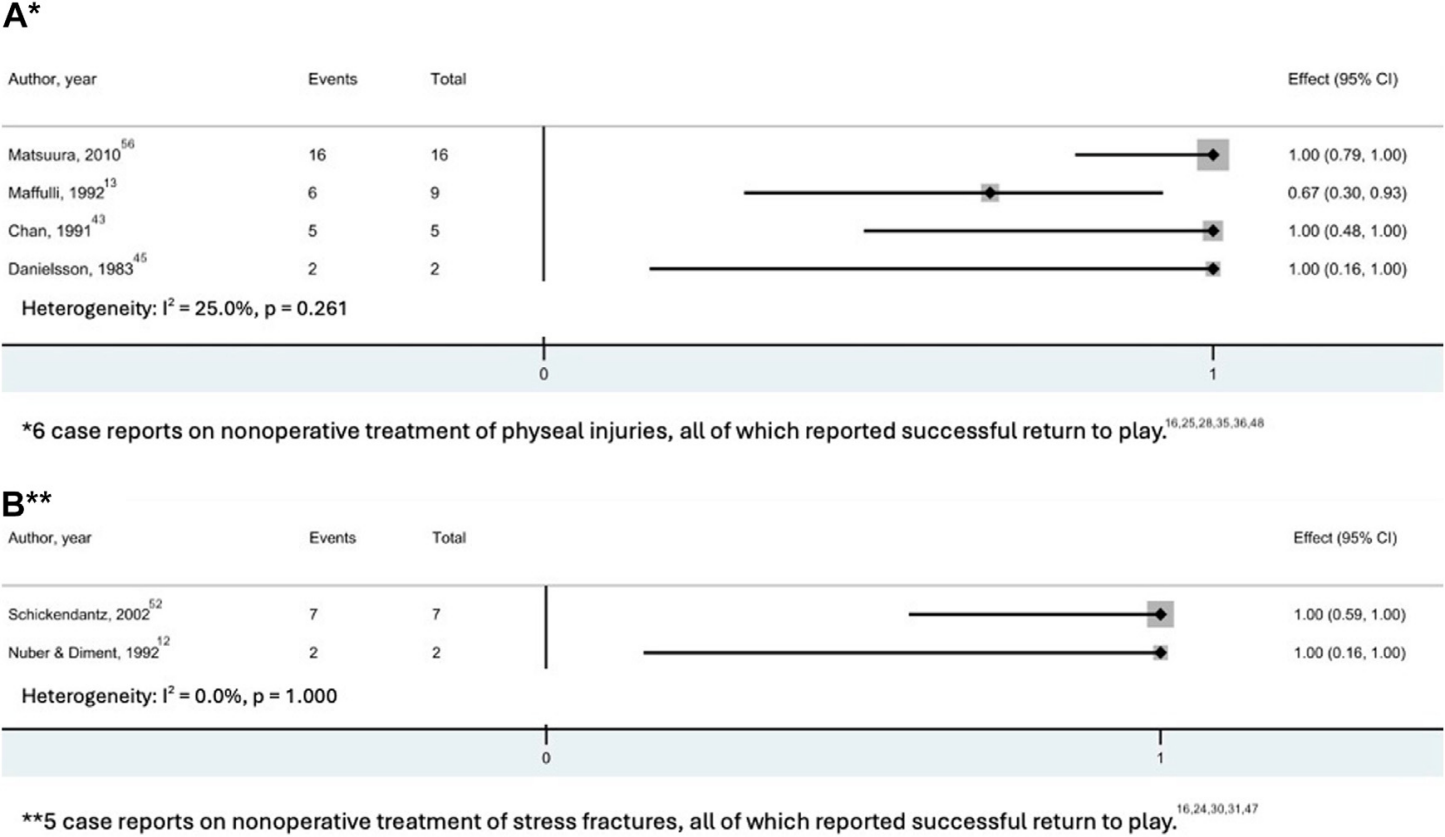
Forest plots for studies reporting on return to play after nonoperative treatment of physeal injuries (A) and stress fractures (B). (CI, confidence interval.)

**Fig 5:**
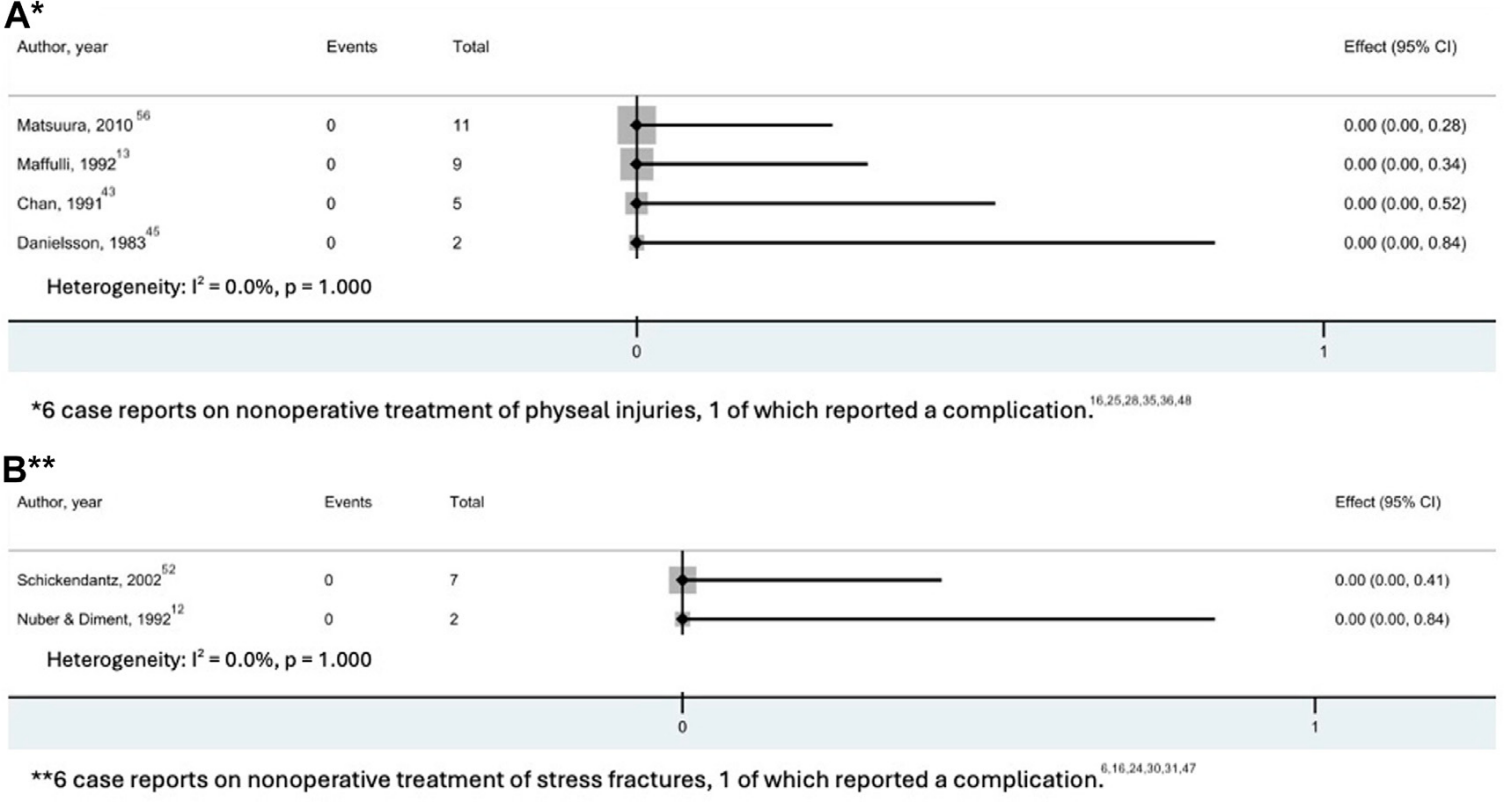
Forest plots for studies reporting on complications after nonoperative treatment of physeal injuries (A) and stress fractures (B). (CI, confidence interval.)

The outcomes of nonoperative treatment of olecranon stress injuries were reported by 17 studies, including 48 athletes (Table 2). The age range of patients undergoing nonoperative treatment was 11 to 28 years (median, 15 years [IQR, 13-17 years]). Nonoperative treatments included rest, bracing with a hinged elbow orthosis, physical therapy, ultrasound therapy, and posterior mold splinting. The duration of nonoperative treatment ranged between 8 and 26 weeks (median, 12 weeks [IQR, 9.5-13 weeks]). Serial radiographic follow-up was reported in 16 athletes. The time to radiographic union ranged between 4 and 18 months (median, 9.4 months [IQR, 6.2-16.6 months]). Studies that reported on return to play are presented in Figure 4. The return to play rates ranged from 67% to 100% (median, 100% [IQR, 100-100%]). The time to return to play was reported in only 8 athletes, ranging between 1 and 9 months (median, 4 months [IQR, 2.8-5.3 months]) after injury. Three gymnasts gave up sport because of injuries unrelated to the stress injury.^13^ Complication rates ranged between 0 and 100% (median, 0% [IQR, 0-0%])^28^^,^^30^ (Fig 5). The complications occurred in 2 case reports and included chondromalacia of the trochlear notch (n = 1) and “occasional symptomatology” (n = 1).

**Table 2:** Summary of Nonoperative Outcomes

Study	Sample size, n	Treatment details	Treatment Duration, Mean (Range), wk	Time to Radiographic Healing, Mean (Range), wk	Time to Radiographic Union, Mean (Range), wk
Physeal injuries (n = 33)					
Botwin et al.^25^ (2018)	1	Rest, low-intensity pulsed ultrasound	8	22	30
Matsuura et al.^56^ (2010)	11	Rest	13 (NR)	NR	19 (NR)
Shinozaki et al.^36^ (2006)	1	Rest	9	9	NR
Parr and Burns^16^ (2003)	1	Avoiding heavy use	9	13	NR
Lowery et al.^48^ (1995)	1	Rest, stretching	13	13	26
Maffulli et al.^13^ (1992)	9	Rest, cryotherapy, PT	NR	NR	NR
Chan et al.^43^ (1991)	5	NR	NR	NR	NR
Retrum et al.^35^ (1986)	1	Rest	26	NR	157
Danielsson et al.^45^ (1983)	2	Rest	13 (NR)	NR	NR
Hunter and O’Conner^28^ (1980)	1	Rest	8	43	NR
Stress fractures (n = 15)					
Brucker et al.^6^ (2015)	1	Rest, throwing program	12	NR	NR
Blake et al.^24^ (2008)	1	Rest, electronic stimulator, PT	22	NR	NR
Lu et al.^30^ (2006)	1	Rest	NR	NR	52
Parr and Burns^16^ (2003)	1	Rest, ice, NSAID	22	NR	NR
Schickendantz^52^ et al. (2002)	7	Orthosis, progressive rehabilitation	12 (NR)	NR	NR
Mamanee et al.^31^ (2000)	1	Rest, throwing program	12	NR	NR
Nuber and Diment^12^ (1992)	2	Splint, rest, ROM exercises	11 (9 - 13)	NR	NR
Hulkko et al.^47^ (1986)	1	Rest	“Several months”	NR	78

NR, not reported; NSAID, nonsteroidal anti-inflammatory drugs; PT, physical therapy; ROM, range of motion.

### Operative Treatment

The outcomes of operative treatment of olecranon stress injuries were reported in 29 studies, including 126 athletes (Table 3). The age range of patients undergoing operative treatment was 11 to 30 years (median 16 [IQR 15-20 years]). Operative treatment was indicated for 61 athletes because if the failure of nonoperative treatment, which occurred between 1 and 35 weeks (median, 13 weeks [IQR, 7.3-24 weeks]). Surgical procedures involved internal fixation using one cannulated screw, tension banding system with Kirschner wires, bone grafting, and fragment excision. Postoperative protocols were described in 39 of 126 athletes (31%) and included arm casting or splinting, range of motion exercises, and gradual return to throwing programs.

**Table 3:** Summary of Operative Outcomes

Study	Sample size, n	Failed Nonoperative Treatment	Treatment Duration, Mean (Range), wk	Time to Radiographic Healing, Mean (Range), wk	Time to Radiographic Union, Mean (Range), wk
Physeal injuries (n = 46)					
Yamaura et al.^54^ (2018)	2∗	Yes	IF: tension band (2), screw (1)	26 (26-26)	16 (NR)
Frank et al.^7^ (2017)	12	Yes	IF: tension band (11), screw (1)	NR	8 (NR)
Mauro et al.^32^ (2011)	1	Yes	IF: screw	17	NR
Clark and McKinley^27^ (2010)	1	No	IF: plate and bone grafting	6	NR
Matsuura et al.^56^ (2010)	5	Yes	IF: tension band	NR	19 (NR)
Rettig et al.^51^ (2006)	5	Yes (4/5)	IF: screw (4), tension band (1)	10 (10-10)	15 (6.1-33)
Burman et al.^26^ (2004)	1	No	Fragment excision	NR	NR
Parr and Burns^16^ (2003)	1	No	NR	NR	17
Charlton and Chandler^44^ (2003)	5	Yes	IF: tension band	17 (17-17)	9 (NR)
Rao et al.^34^ (2001)	1	No	IF: tension band	NR	17
Tavares^38^ (1998)	1	No	IF: tension band	NR	30
Lowery et al.^48^ (1995)	2	No	IF: tension band	9 (9-9)	17 (1/2)
Walker^40^ (1995)	1	Yes	IF: tension band	NR	104
Maffulli et al.^13^ (1992)	1	Yes	IF: screw	NR	NR
Chan et al.^43^ (1991)	2	NR	IF: not specified	NR	NR
Wilkerson and Johns^59^ (1990)	1	Yes	IF: tension band	NR	52
Weiss and Sauers^41^ (1990)	1	Yes	IF: tension band	NR	22
Pavlov et al.^50^ (1981)	2	No	IF: screw (1); AGB (1)	NR	13 (1/2)
Torg et al.^39^ (1977)	1	No	IF: ABG	NR	56
Stress fractures (n = 80)					
Shanley et al.^57^ (2023)	1	No	NR	NR	NR
Zaremski et al.^58^ (2022)	1	Yes	IF: not specified	35	NR
Erickson et al.^55^ (2019)	46	No	IF: 1 screw (31), multiple screws (8), plate (4), tension band (3)	NR	NR
Paci et al.^49^ (2013)	18	Yes	IF: screw	16 (NR)	11 (3.9-92.9)
Stephenson et al.^37^ (2012)	1	No	IF: screw	16	NR
Fujioka et al.^46^ (2012)	6	Yes	IF: screw	NR	NR
Imade et al.^29^ (2011)	1	No	IF: cable pin system	9	52
Nakaji et al.^33^ (2006)	1	Yes	IF: tension band	11	11
Suzuki et al.^53^ (1997)	2	Yes	IF: screw	NR	52 (1/2)
Hulkko et al.^47^ (1986)	3	No	IF: tension band (2); Fragment excision (1)	NR	17 (1/3)

ABG, autograft bone grafting; IF, internal fixation; NR, not reported.

∗ One patient had bilateral physeal fractures, both requiring operative management.

**Fig 6:**
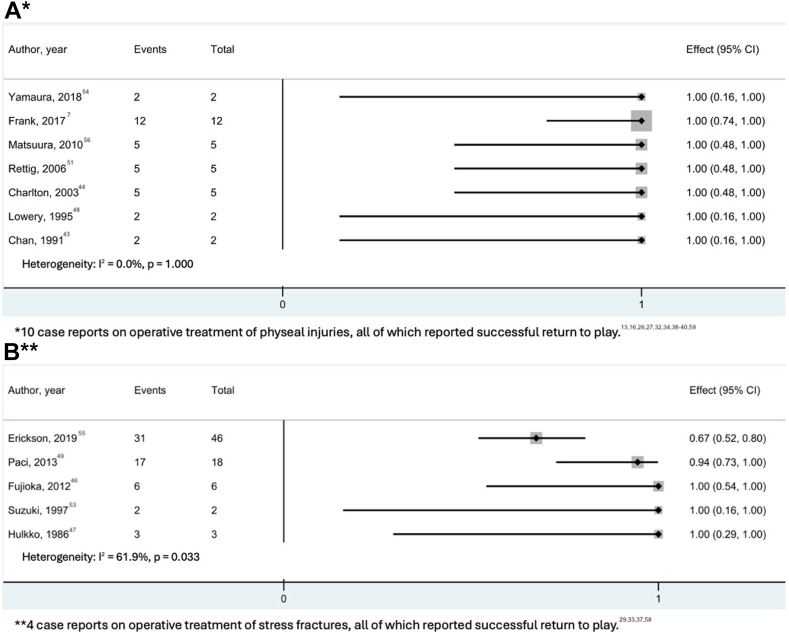
Forest plots for studies reporting on return to play after operative treatment of physeal injuries (A) and stress fractures (B). (CI, confidence interval.)

**Fig 7:**
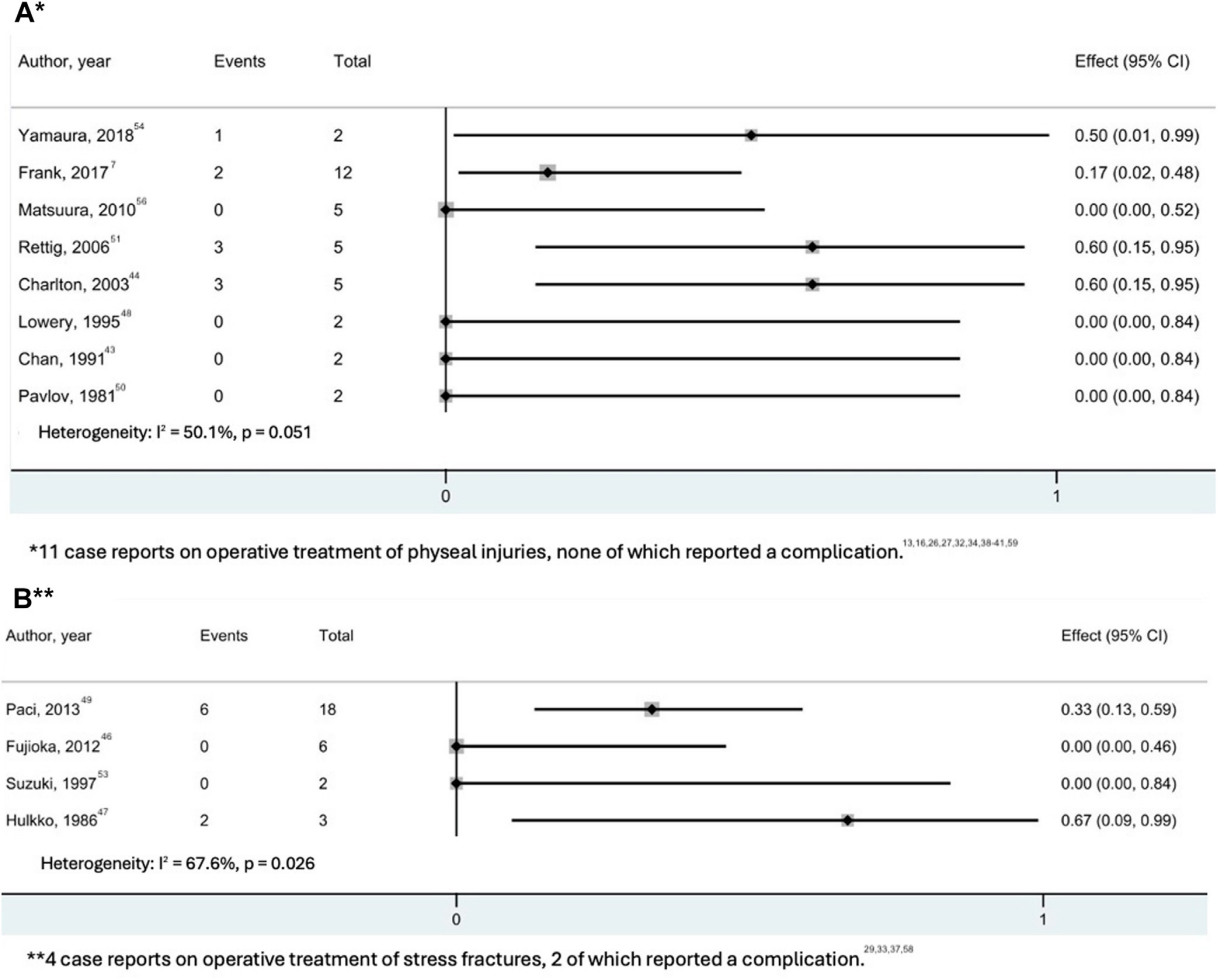
Forest plots for studies reporting on complications after operative treatment of physeal injuries (A) and stress fractures (B). (CI, confidence interval.)

Radiographic follow-up was reported for 60 athletes. The time to radiographic union ranged between 1 and 24 months (median, 17 weeks [IQR, 14-41 weeks]) (Table 3). Studies that reported on return to play are presented in Figure 6. The return to play rates ranged from 67% to 100% (median, 100% [IQR, 100-100%]). The time to return to play ranged between 3 and 18 months (median, 6 months [IQR, 4.5-6.8 months]) after injury. Return to play was described as returning to a previous level of activity for 24 studies and not specified in five.^34^^,^^40^^,^^41^^,^^46^^,^^57^ Of the 20 athletes who failed to return to play, 15 were attributable to the high demands of pitching in professional baseball, one because of persistent elbow pain, and 4 patients did not specify a reason.^41^^,^^50^^,^^55^ Complication rates ranged between 0 and 100% (median, 0% [IQR, 0-50%]). The complications occurred in 8 studies (2 case reports) and included hardware irritation (n = 15), recurrence of the stress fracture (n = 3), fracture displacement requiring regrafting (n = 1), and ulnar nerve impingement requiring surgical release (n = 1) (Fig 7).

## Discussion

Most athletes (126/174; 72%) underwent operative treatment, with almost one half of the included studies (15/40) reporting athletes were nonresponsive to an initial trial of nonoperative management and were converted to operative management. In both treatment groups, patients returned to sport within similar time frames. These findings confirm the hypothesis that most athletes would undergo operative management. The most common complications after operative management were hardware irritation and recurrence of the stress fracture. Patients most commonly presented with elbow tenderness to palpation or swelling. Plain radiographs were sufficient to diagnose the majority of physeal injuries, with 3 athletes requiring MRI and 1 a CT scan. For stress fractures, MRI was necessary for 35 athletes and CT scan for 2 to confirm the diagnosis.

This systematic review adds to existing evidence on the management of proximal ulna stress injuries by including athletes from a range of sports. To date, there is limited literature addressing this topic. Smith et al.^14^ conducted a systematic review focusing on stress fractures of the elbow in 52 patients across 14 studies. However, their analysis specifically examined management and outcomes in overhead-throwing athletes and included injuries to the distal humerus, which were not within the scope of our study. Additionally, data on imaging modalities used in previous studies was collected to elucidate anatomical variation (Figs 2 and 3). As a result, all studies including nonrandomized series were analyzed. Inclusion of these studies introduces selection bias and confounding factors, which may lead to misinterpretation. To address this, pooling of heterogenous data was avoided and quality assessment tools were used to assess the included studies.

### Clinical Recommendations

The diagnosis of an olecranon stress injury should be considered in athletes with posterior elbow pain during athletic activity with the absence of pain during rest and without symptoms of instability. Symptoms in young athletes (<20 years of age) may arise from delayed physeal closure or the persistence of the olecranon physis, given that the growth plate in the olecranon is among the last to close in the elbow.^60^ Complete fusion is expected at approximately 16 years of age for male athletes and 13 years old for female athletes.^61^ In older athletes, there is a greater suspicion for olecranon stress fractures. Two classification systems have been proposed for proximal ulna stress injuries, including both physeal injuries and stress fractures, on the basis of the direction of the fracture line and age.^10^^,^^11^ The results of the present study align with a range of the injury subtypes from both classifications and support their findings.

During physical examination, the elbow should be assessed for swelling and tenderness to palpation of the posterior, posteromedial, and/or posterolateral proximal ulna. Elbow range-of-motion deficits seem to be frequently observed, more commonly in athletes with physeal injuries than stress fractures. Tests that elicit valgus and/or traction forces on the proximal ulna (e.g., valgus stress testing, forced hyperextension, or resisted elbow extension) may reproduce pain, but distinguishing symptoms from other elbow pathologies such as UCL injury is important. Because of frequent concomitant pathology, evaluation of the integrity of the UCL is recommended.^11^^,^^32^ Furthermore, glenohumeral rotation deficits of the shoulder should be assessed, as these might be related to stress fractures of the ulna. In addition identified patient-specific risk factors, calcium, iron, and vitamin D levels may influence the risk of stress fractures and should therefore be monitored in athletes.^62^^,^^63^ Only one^37^ of the 40 studies covered in this review reported the assessment of the athlete’s endocrinological/nutritional status.

When assessing for stress injuries, bilateral plain radiographs are the standard choice of imaging. These radiographs may reveal signs such as delayed or widened closure of the physis when compared with the contralateral side. Previous studies suggest that these signs on plain radiographs in combination with sclerotic margins are associated with poor outcomes of nonoperative management in athletes.^7^^,^^13^^,^^43^^,^^44^^,^^51^^,^^56^ In cases in which plain radiographs yield inconclusive results, MRI and CT scans are used for diagnosis. MRI is preferred for its ability to detect stress fractures early, precise localization of injuries, and lack of ionizing radiation.^42^^,^^64^, ^65^, ^66^ On MRI, the integrity of other structures at risk for injury in elbow-loading athletes may also be assessed, including the UCL and radiocapitellar articulation. In cases in which incomplete fracture lines are present, indicative of nondisplaced stress fractures or osseous stress reactions, CT scans should be used to detect specific fracture patterns and assess if additional fractures are present.^67^

Nonoperative management is recommended in athletes with nondisplaced symptomatic physeal widening without sclerotic margins or nondisplaced stress fractures. This should include a period of relative rest, cessation of athletic activity involving the symptomatic elbow, range-of-motion and strengthening exercises, and a gradual return to athletic activity. In high-demand athletes, supervision of return-to-play programs is recommended. Our results suggest that the mean duration of nonoperative management before transitioning to operative management was 15 weeks. Therefore, it is crucial to counsel athletes and manage their expectations regarding the timeline for full healing. In athletes with osseous stress injury of the proximal ulna, MRI may show resolution of bone marrow edema as early as 4 weeks after diagnosis, but it has been suggested that these findings poorly correlate with patients’ symptoms.^52^ Therefore, it is recommended to determine return to play decision making on clinical presentation rather than MRI findings in athletes with this type of stress injury.

Fixation may be required in athletes with limited or no bone healing, the emergence of sclerotic margins of the widened physis, or persistent stress fractures. A previous study on stress fractures of the elbow in throwing athletes reported tension banding and/or cannulated screw fixation to be the most common constructs used to treat these injuries, with a 96% return-to-sport rate at or above their preinjury level.^14^ Athletes should follow a gradual postoperative protocol for return to play, including a period of rest for soft tissue healing (with or without splinting), range of motion exercises, and strengthening exercises (e.g., Thrower’s Ten program for throwers). Bony union can be expected around 4 months postoperatively but ranges from 1 to 24 months. Restoration of range of motion to the pre-injury range, no pain with valgus stress testing and/or forced hyperextension, and normal strength of the upper extremity have been proposed as useful return to throwing criteria.^52^ Future research should be prospective and evaluate predictors of treatment switches such as fracture morphology and therapy compliance.

### Limitations

There are a couple of limitations to this study. First, the available literature on proximal ulna stress injuries in athletes used in this study only consists of low-level evidence (Level III-IV). This is likely because of the rarity of this injury, making it challenging to conduct higher-level studies. Second, the inclusion of athletes from any sport, variation of injury location, and nonstandardized treatment options by treating doctors make our data heterogeneous. It is advised to prevent the pooling of data from heterogeneous studies, as confounding factors cannot be accounted for.^20^ Thus, subjective synthesis was used to describe the results and forest plots were generated for outcomes (i.e., return to play and complications).

## Conclusions

Proximal ulna stress injuries in athletes can be managed either nonoperatively or operatively, with both showing satisfactory rates of return to play. Although operative management was more common, almost half of these athletes failed an initial trial of nonoperative management.

## Disclosures

The authors declare the following financial interests/personal relationships which may be considered as potential competing interests: M.P.J.v.d.B. reports funding grants from 10.13039/100009026Smith & Nephew. T.V.L. reports consulting or advisory for DePuy Synthes and speaking and lecture fees from the 10.13039/501100001702AO Foundation. All other authors (J.S.B., R.J.M., H.H.d.K., B.B., J.N.D., D.E.) declare that they have no known competing financial interests or personal relationships that could have appeared to influence the work reported in this paper.
